# Histopathological Changes Caused by Inflammation and Oxidative Stress in Diet-Induced-Obese Mouse following Experimental Lung Injury

**DOI:** 10.1038/s41598-018-32420-3

**Published:** 2018-09-24

**Authors:** Fengyuan Wang, Zhicai Zuo, Kejie Chen, Jing Fang, Hengmin Cui, Gang Shu, Yi Zhou, Zhengli Chen, Chao Huang, Wentao Liu

**Affiliations:** 1 College of Veterinary Medicine, Sichuan Agricultural University, Chengdu, Sichuan 611130 PR China; 2 School of Public Health, Chengdu Medical College, Chengdu, Sichuan 610500 PR China; 3 Life science department, Sichuan Agricultural University, Yaan, Sichuan 625014 PR China

## Abstract

Obesity has been identified as a risk factor for adverse outcomes of various diseases. However, information regarding the difference between the response of obese and normal subjects to pulmonary inflammation is limited. Mice were fed with the control or high-fat diet to establish the lean and diet-induced obese (DIO) mice. *Escherichia coli* was intranasally instilled to reproduce non-fatal acute pneumonia model. After infection, serum samples and lung tissues were obtained at 0, 12, 24, and 72 h. DIO mice exhibited increased serum triglyceride (TG) and total cholesterol (TC) contents as well as pulmonary resistin, IL-6, and leptin levels compared with lean mice. *E. coli* infection caused an acute suppurative inflammation in the lung with increased lung index and serum TG and TC contents; elevated pulmonary tumor necrosis factor-α, interleukin (IL)-1β, IL-6, IL-8, and leptin levels; and oxidative stress in mice. Interestingly, almost all the above-mentioned parameters peaked at 12 h after infection in the lean-*E. coli* group but after 12 h in the DIO-*E. coli* group. These results indicated that the DIO mice presented a delayed inflammatory response and oxidative stress in non-fatal acute pneumonia induced by *E. coli* infection.

## Introduction

The lungs are the primary organ of the respiratory system in humans and many other animals. They are responsible for gas exchange in the respiratory system. The elasticity and tensile strength of the lungs are ensured by their connective tissue network^1^. Mucins (glycosylated proteins) produced by epithelial tissues in the air conduction part of the lungs play an important role in defense against foreign bodies and pathogenic microorganisms and are overexpressed in several lung diseases^2^.

*Escherichia coli*, a gram-negative bacillus, is a kind of respiratory pathogen and strong inducer of pro-inflammatory cytokine, and it can cause nosocomial pneumonia^3^. Moreover, sublethal intratracheal challenges of *E. coli* in mice result in the migration of neutrophils into the pulmonary air space^4^. Neutrophils that infiltrate the lungs and migrate into the airways express pro-inflammatory cytokines, such as interleukin (IL)-1β and tumor necrosis factor (TNF)-α, and appear to contribute to oxidant-induced injury and loss of epithelial integrity^5^.

Obesity is a medical condition in which excess body fat has accumulated to the extent that may pose a negative effect on health. Worldwide obesity has nearly tripled since 1975^6^. Obesity increases morbidity and mortality from many chronic health ailments, such as cardiovascular disease, type II diabetes, dyslipidemia, and fatty-liver disease^7^. Obesity also increases the risk of respiratory tract infections, such as pneumonia^8^. However, paradoxically, recent studies reported that obese patients show improved outcome such as reduced mortality in acute bacterial pneumonia^9–11^. In obesity, the ability of the adipose tissue to elaborate cytokines and adipocytokines, such as IL-6, TNF-α, leptin, and resistin, is increased to possess pro-inflammatory properties. The low-grade chronic systemic inflammation associated with obesity can potentially enhance pulmonary immune responses against respiratory infections^12^.

The reasons of these findings are unclear. One potential factor is the alteration of pulmonary immune responses in the obese, which may lead to a heightened state of host defense^13^. It will be enthralling to shed light on how the lung is affected by acute bacterial pneumonia in obese models from earlier reports of pneumonia. To understand this concept, we selected ICR mice fed with high-fat diet to establish the obese model. We instilled *E. coli* intranasally in mice to establish an acute nonfatal bacterial pneumonia model. Subsequently, changes in histopathology and cytokines, adipocytokine secretion, and oxidative stress status were detected to investigate the impact of obesity on nonfatal pneumonia induced by *E. coli*.

## Results

### Increased body weight of ICR mice with high-fat diets

**Figure 1:**
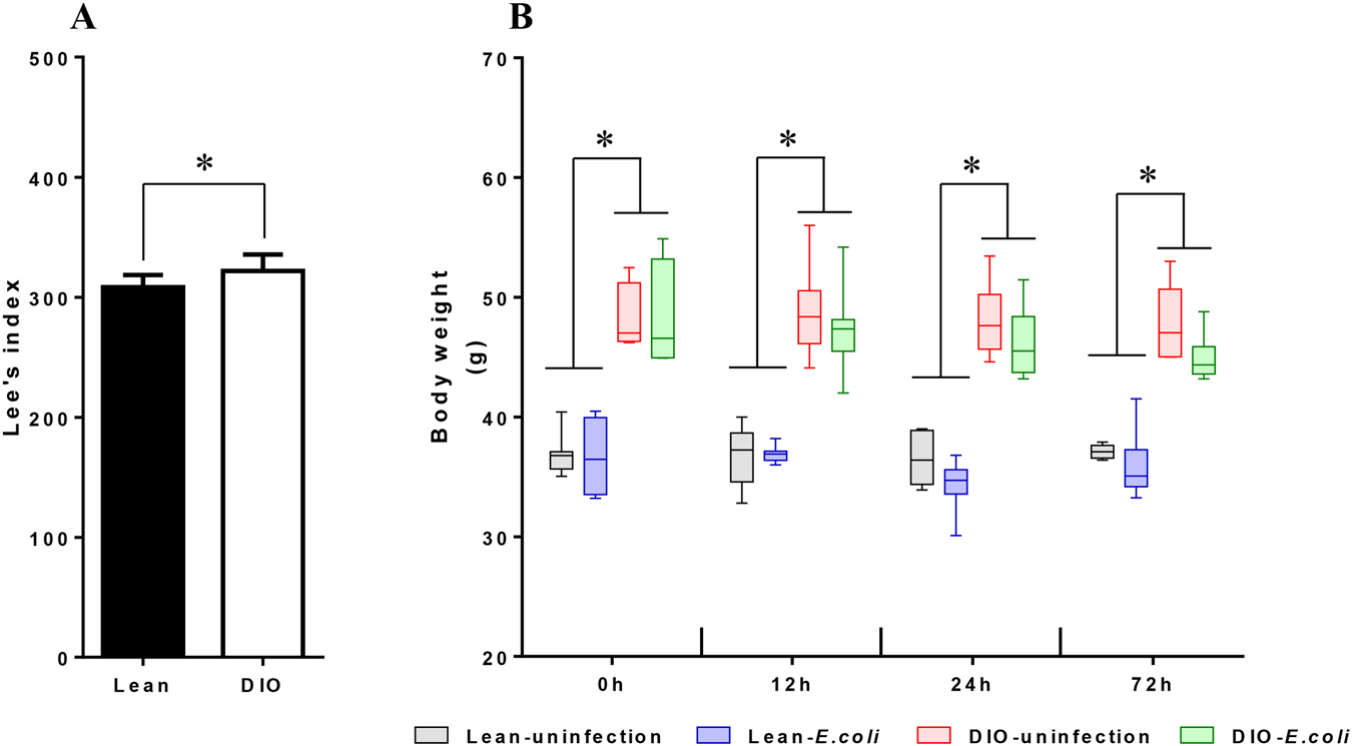
Lean and DIO ICR mice underwent acute pulmonary *E. coli* infection. (**A**) Lee’s index at baseline (**B**) Body weight at baseline and at different time points after *E. coli* instillation. ^*^p < 0.05.

Lee’s index of DIO mice was significantly higher (*p* < 0.05) than that of the lean mice. During the experiment, the body weight of the DIO groups were higher than that of the lean groups (*p* < 0.05). After infection, the body weight in the lean-*E. coli* group and the DIO-*E. coli* group showed a decreasing tendency compared with the uninfected groups (Fig. 1).

### Changes of serum triglyceride (TG) and total cholesterol (TC) in mice following *E. coli* infection

**Figure 2:**
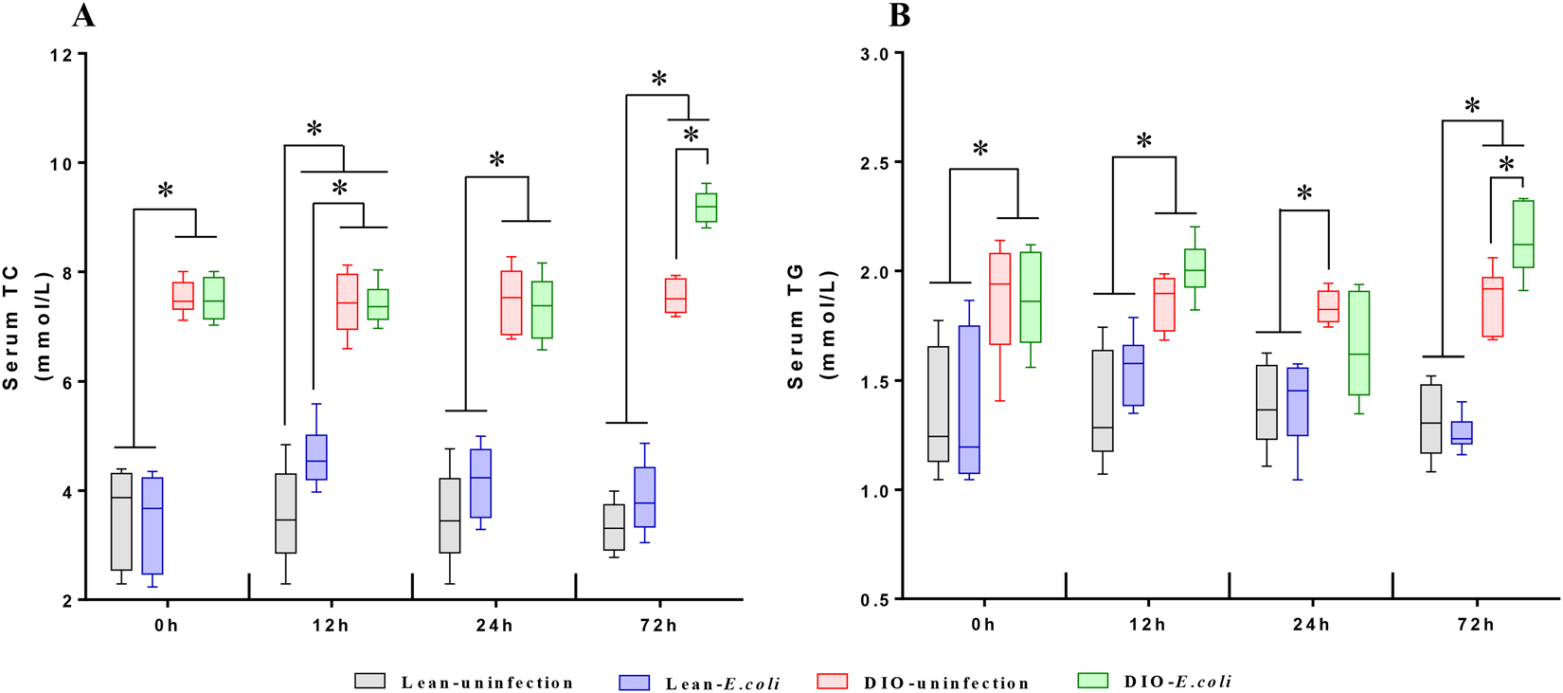
Serum triglycerides (**A**) and total cholesterol (**B**) levels of lean and obese mice at different time points following *E. coli* infection. ^*^p < 0.05.

As shown in Fig. 2, at 0 h, the DIO mice exhibited markedly higher serum TG and TC contents than the lean mice (*p* < 0.05). After being infected with *E. coli*, the serum TG and TC contents in the DIO-*E. coli* group significantly increased at 72 h compared with the DIO-uninfected group. In the lean-*E. coli* group, only the serum TC content markedly increased at 12 h in comparison to the lean-uninfected group (*p* < 0.05).

### Change in lung weight and lung index following *E. coli* infection

**Figure 3:**
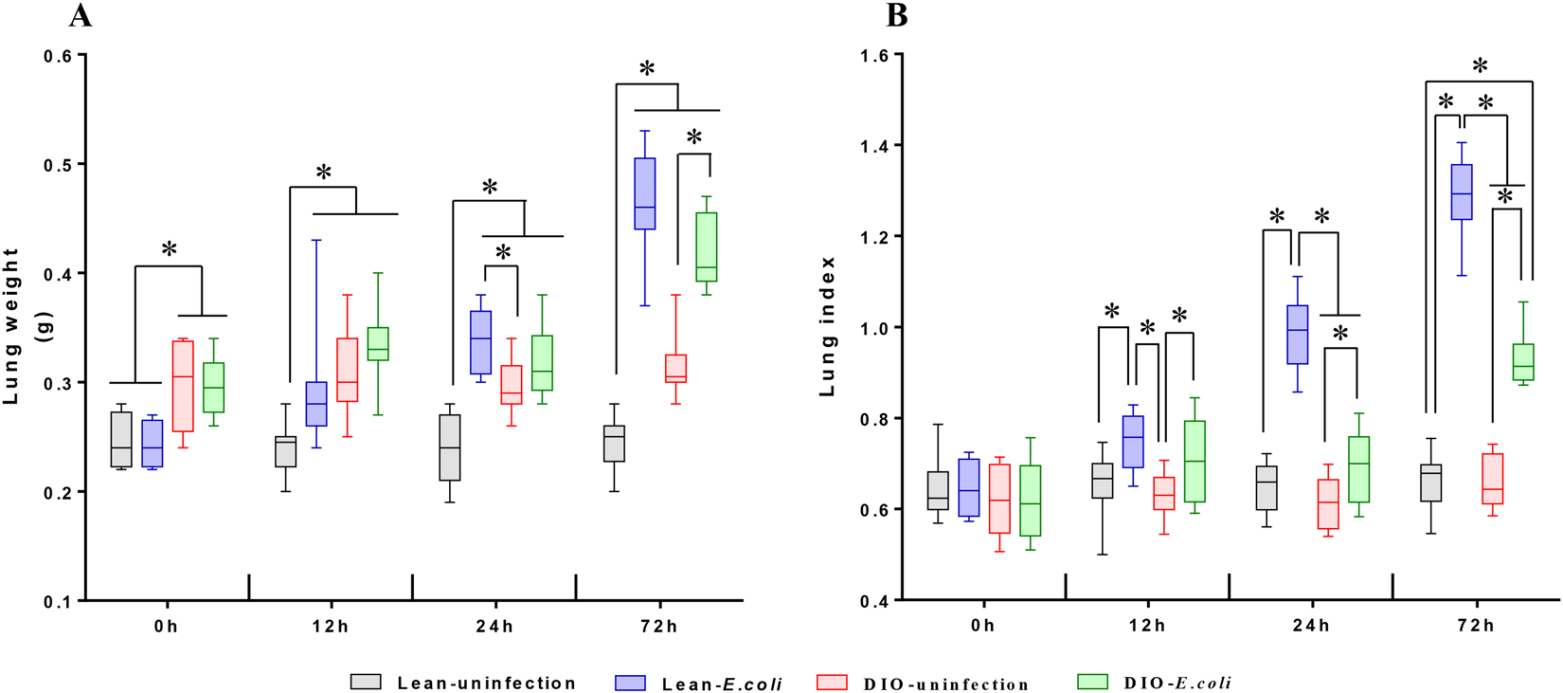
Lung weight (**A**) and lung index (**B**) of lean and obese mice at different time points following *E. coli* infection. ^*^p < 0.05.

Before being treated with *E. coli*, the lung weight was higher in the DIO group than in the lean group (*p* < 0.05). After being infected with *E. coli*, the lung weight significantly increased in the lean-*E. coli* group compared with the lean-uninfected group from 12 h to 72 h. Interestingly, we only detected a significant increase (*p* < 0.05) of lung weight in the DIO-*E. coli* group at 72 h in comparison to the DIO-uninfected group. Moreover, the lung index showed no significant difference at 0 h (*p* > 0.05). During the whole 72 h post-infection, increased lung index was detected in the *E. coli*-infected groups compared with the uninfected groups (*p* < 0.05) (Fig. 3).

### Pathological injuries of the lung following *E. coli* infection

**Figure 4:**
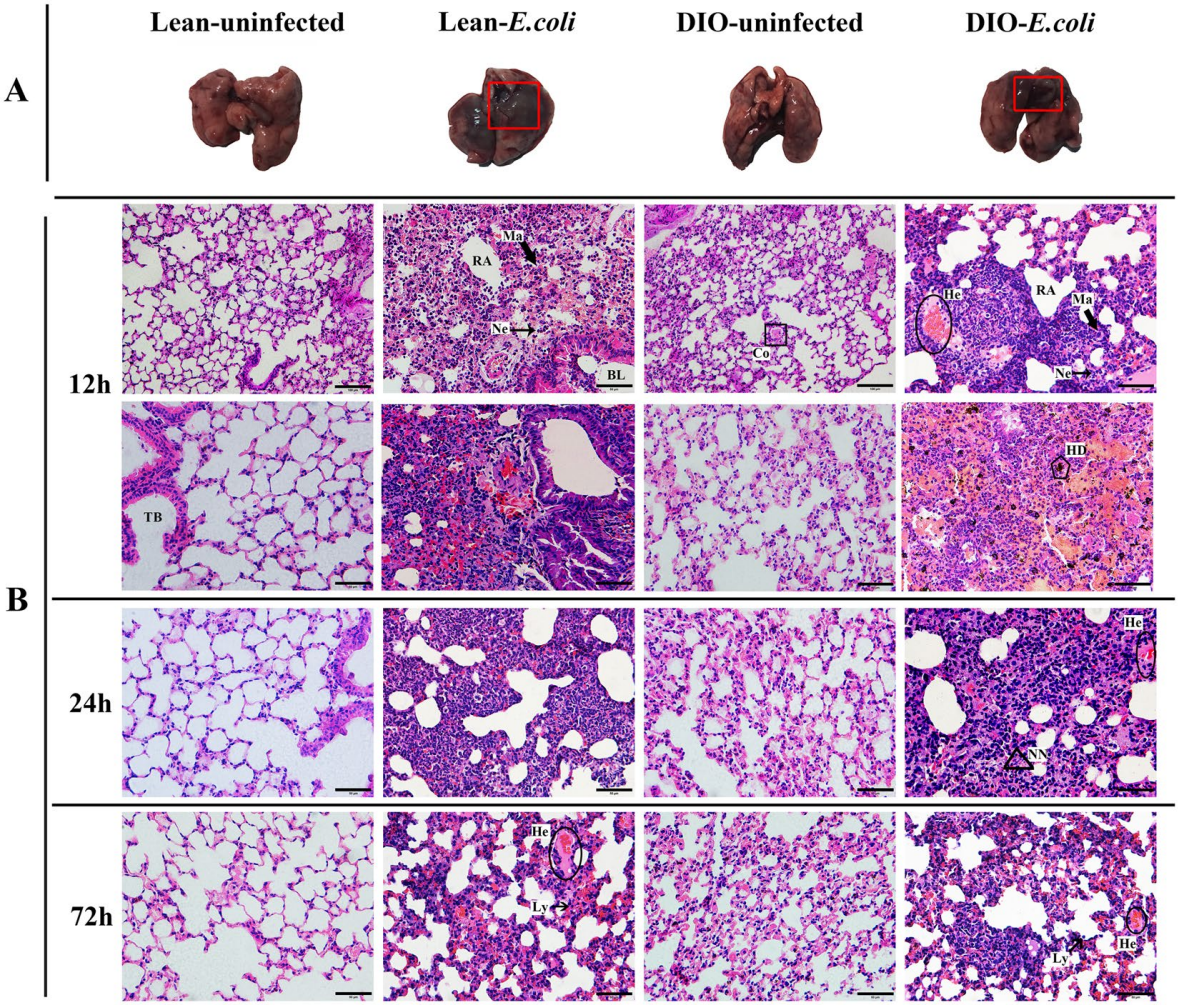
(**A**) Representative lung anatomy changes following *E. coli* (12 h). Infection-affected areas are indicated by red boxes. (**B**) Pulmonary histopathology of lean and obese mice after *E. coli* infection (12, 24 and 72 h) by Hematoxylin-eosin staining. Scale bar: 50 μm (High magnification) or 100 μm (Low magnification). BL (bronchiole lumen); TB (terminal bronchiole); RA (residual alveolus); Ma (pulmonary macrophage); Ne (neutrophil); Ly (lymphocyte); NN (necrotic neutrophil); Co (Congestion); He (hemorrhage); HD (hemosiderin deposition).

Figure 4 shows that the lungs with uniform pink color in the lean- and DIO-uninfected groups exhibited normal macroscopic structure. After being infected with *E. coli*, the texture of the affected areas in the superior lobes of the left and right lungs became hard with a dark red or grey appearance. The affected area in the DIO-*E. coli* group was smaller than that in the lean-*E. coli* group.

Before infection, the normal histology of conducting airway structure (bronchi and bronchioles) and gas exchange area (respiratory bronchioles, alveolar ducts, alveolar sacs, and alveolus) was microscopically observed in each group. As shown in Table 1, compared with the lean-uninfected group, the alveolar wall was thickened with decreased airspace areas, and the incidence of congestion in alveolus and respiratory bronchioles decreased in the DIO-uninfected group. After *E. coli* infection, typical acute suppurative inflammation was noted in the lean- and DIO-*E. coli* groups, as shown in Fig. 4. Nevertheless, the severity of neutrophil infiltration and decrease in the airspace area proportion in the DIO-*E. coli* group were lesser than that in the lean-*E. coli* group. However, serious congestion and hemorrhage in alveolus occurred in the DIO-*E. coli* group compared with the lean-*E. coli* group, which even caused the hemosiderin granules to deposit in the hemorrhage areas. At 24–72 h after infection, the severity of neutrophil infiltrate condition and incidence of congestion and hemorrhage were decreased in the lean- and DIO-*E. coli* group.

**Table 1:** Histologic scoring of pulmonary inflammation.

Time	Groups	Incidence of Congestion	Incidence of Hemorrhage	Severity of Neutrophil infiltrate	Proportion of Airspace area
0 h	Lean-uninfected	1/8	0/8	—	59.85%
	Lean-*E. coli*	1/8	0/8	—	58.66%
	DIO-uninfected	3/8	0/8	—	40.75%
	DIO-*E. coli*	3/8	0/8	—	42.34%
12 h	Lean-uninfected	2/8	0/8	—	73.60%
	Lean-*E. coli*	6/8	4/8	++++	21.52%
	DIO-uninfected	3/8	0/8	—	49.58%
	DIO-*E. coli*	7/8	5/8	+++	26.22%
24 h	Lean-uninfected	1/8	0/8	—	55.20%
	Lean-*E. coli*	5/8	3/8	+++	23.74%
	DIO-uninfected	2/8	0/8	—	49.75%
	DIO-*E. coli*	6/8	4/8	++	24.23%
72 h	Lean-uninfected	1/8	0/8	—	59.50%
	Lean-*E. coli*	5/8	2/8	++	31.60%
	DIO-uninfected	2/8	0/8	—	39.97%
	DIO-*E. coli*	5/8	3/8	++	30.53%

Note: The same position of the lungs in eight mice was observed through a microscope, and the histological lesion of the lung was evaluated through the incidence of congestion and hemorrhage, severity scoring of neutrophil infiltrate, and proportion of airspace areas. The level of severity was judged from − to ++++, which represented none to severe. The airspace proportion was the ratio of the airspace area and the total area of one view under 400× magnification.

### Changes in pulmonary mucins following *E. coli* infection

**Figure 5:**
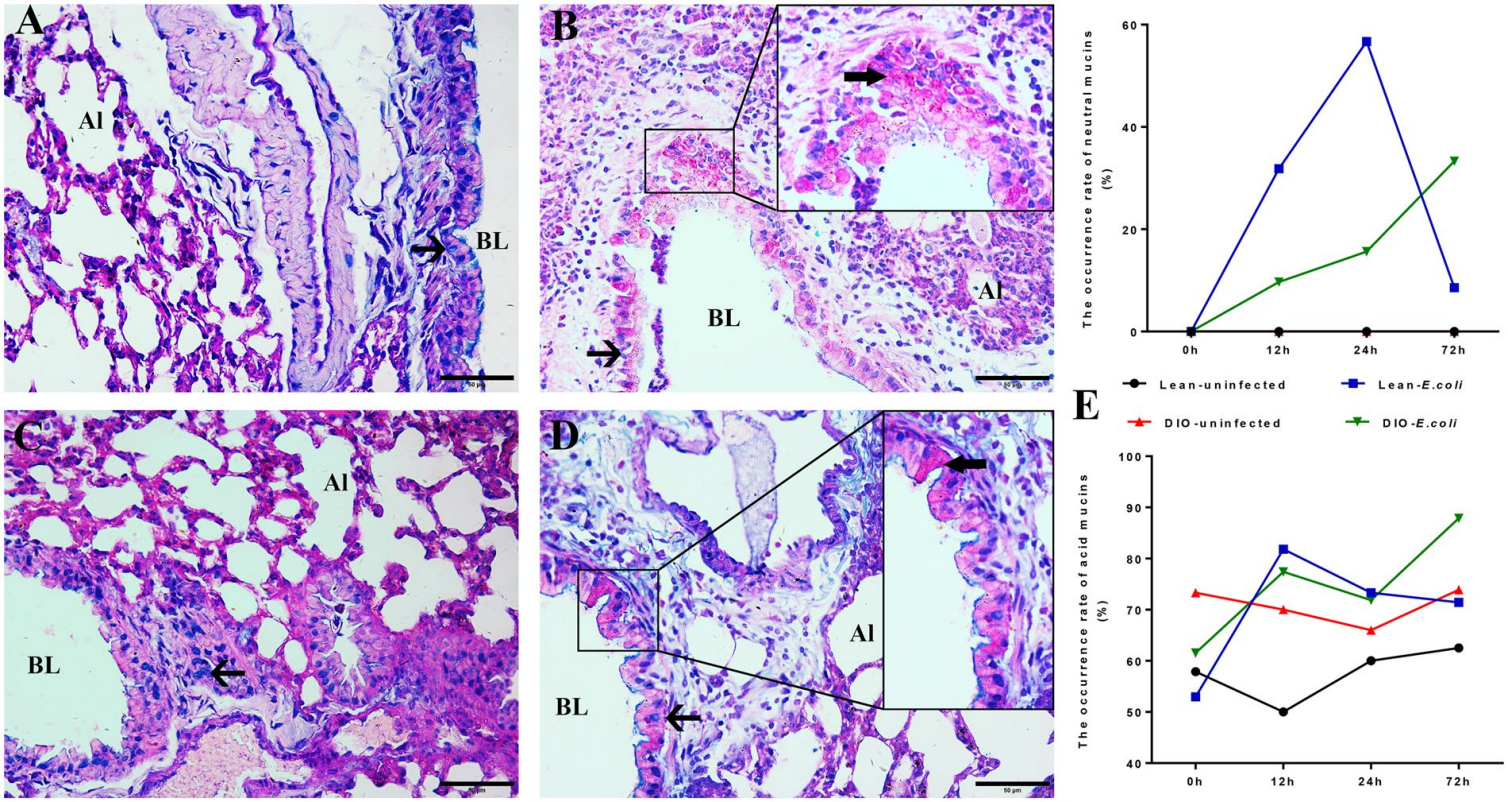
Pulmonary mucins of lean and obese mice following *E. coli* infection (24 h). (**A**–**D**) Representative micrographs of Alcian Blue/PAS staining. (**A**) Lean-uninfected group; (**B**) Lean-*E. coli* group; (**C**) DIO-uninfected group; (**D**) DIO-*E. coli* group. (**E**) Semiquantitative analysis of acid or neutral mucins in intrapulmonary conducting airways. Scale bar: 50 μm. BL (bronchiole lumen); Al (pulmonary alveolus); arrows in enlarged boxes showing neutral mucins (red) and acid mucins (blue).

The mucin occurred in the goblet cell and mucous gland of the respiratory tract. As shown in Fig. 5, acid mucins were observed in the mucosa epithelial cell of intrapulmonary conducting airways (bronchi and bronchiole) in all four groups. The occurrence rate of acid mucins was higher in the DIO groups than in the lean groups before infection. At 12–72 h after infection with *E. coli*, the occurrence rate of acid mucins increased sharply in the lean-*E. coli* group compared with the DIO-*E. coli* group. No neutral mucin was detected in the uninfected groups, whereas the occurrence rate of neutral mucins was increased in the *E. coli*-infected groups. Moreover, the occurrence rate of neutral mucin peaked at 24 h in the lean-*E. coli* group but showed a continuous increasing trend in the DIO-*E. coli* group from 12 h to 72 h.

### Changes in pulmonary collagen fibers following *E. coli* infection

**Figure 6:**
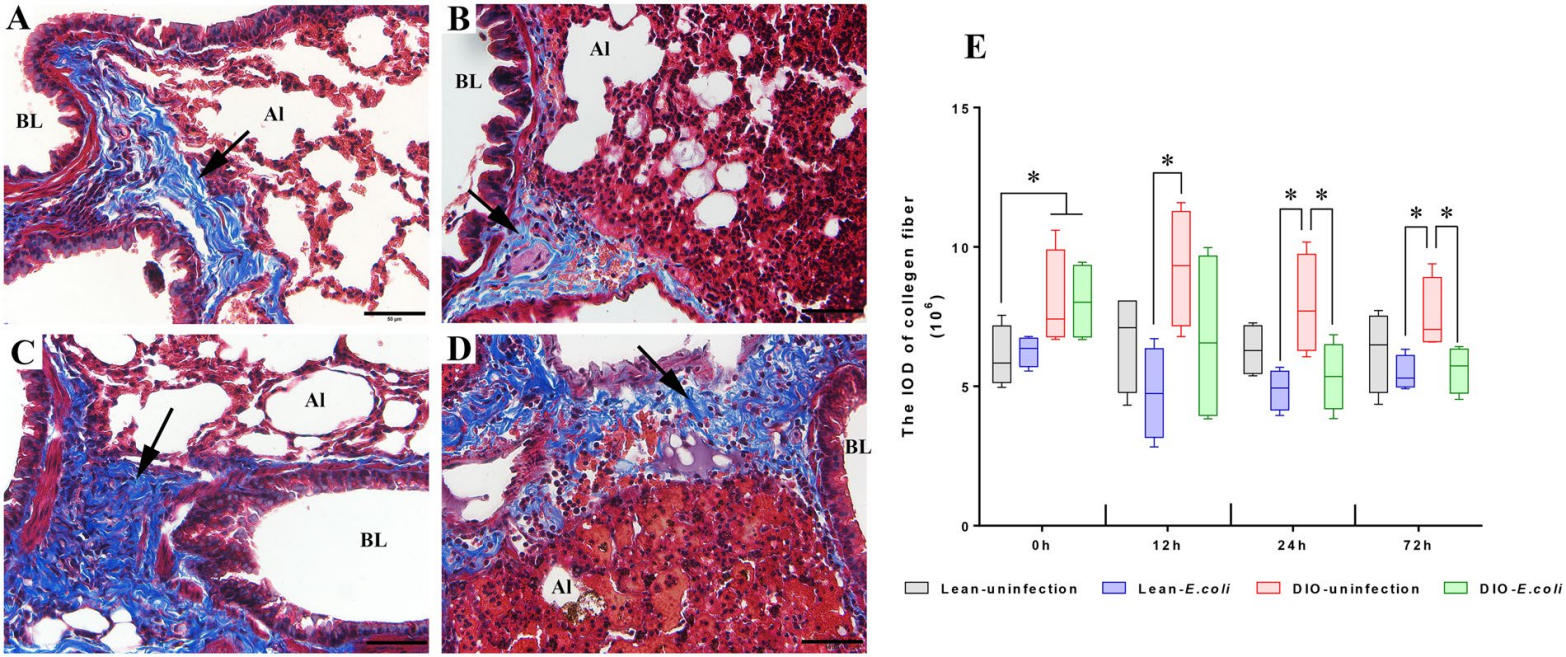
Pulmonary collagen fibers of lean and obese mice following *E. coli* infection (12 h). Representative micrographs of Masson’s trichrome staining, with blue-stained collagen fibers. (**A**) Lean-uninfected group; (**B**) Lean-*E. coli* group; (**C**) DIO-uninfected group; (**D**) DIO-*E. coli* group. (**E**) Semiquantitative analysis of collagen fiber deposition using Integrated Optical Density (IOD) measurement, at different time points after infection. Scale bar: 50 μm. BL (bronchiole lumen); Al (pulmonary alveolus); arrows showing the collagen fibers. ^*^p < 0.05.

The collagen fibers were mainly located in the loose connective tissue below the epithelium and oriented circularly or obliquely around the bronchiole. As shown in Fig. 6, the DIO groups possessed more collagen fibers than the lean groups before infection (*p* < 0.05). After infection, the integrated optical density (IOD) of collagen fibers showed a decreased tendency in the lean-*E. coli* group and DIO-*E. coli* group in comparison to each uninfected group. However, the significant decrease was detected only at 24 and 72 h in the DIO-*E. coli* group (*p* < 0.05).

### Changes in cytokines and adipocytokines following *E. coli* infection

**Figure 7:**
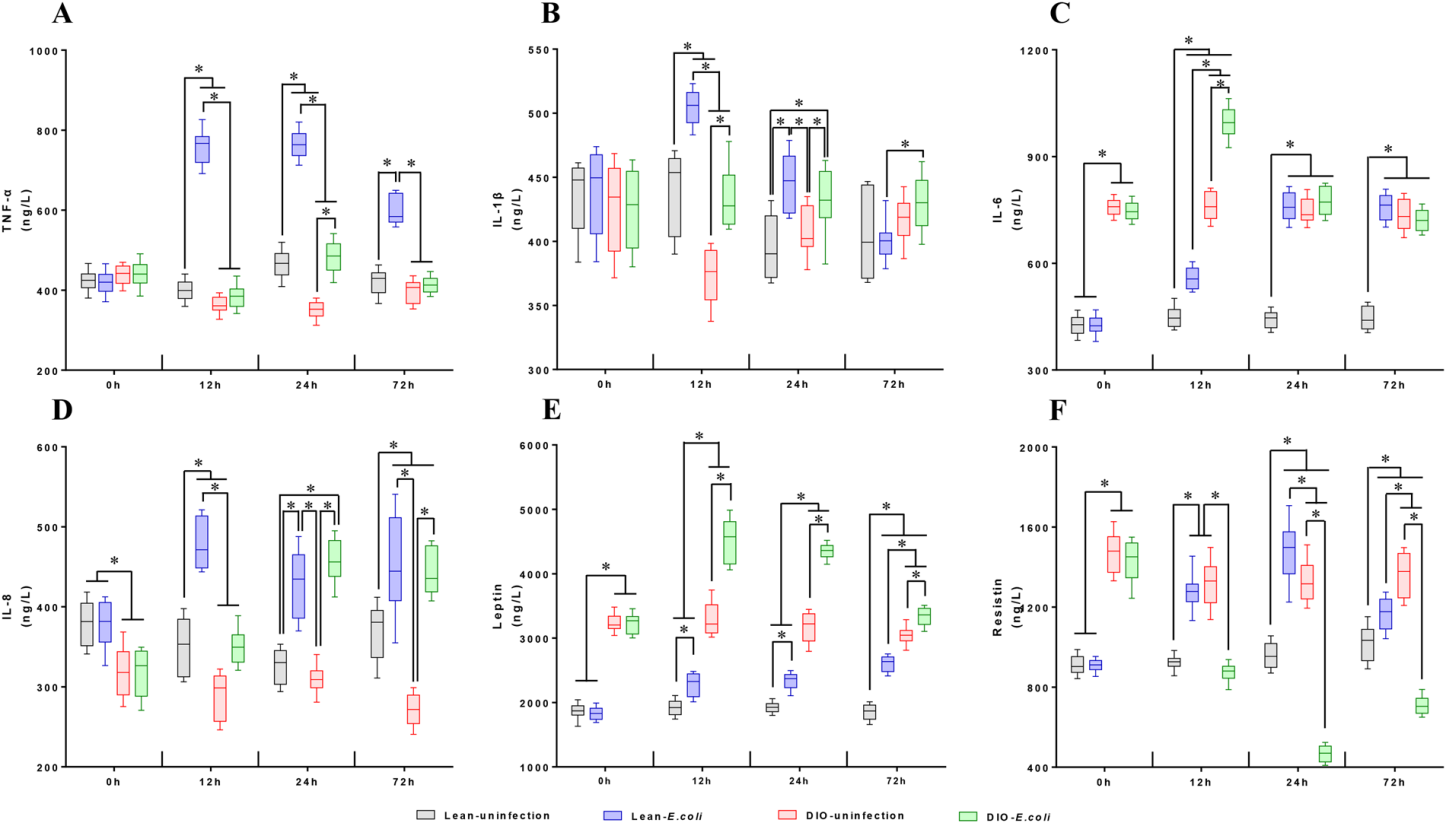
Pulmonary cytokines (**A**–**D**) and adipocytokines (**E**,**F**) in lean and obese mice following *E. coli* infection. ^*^p < 0.05.

The contents of pulmonary cytokines and adipocytokines are shown in Fig. 7. Before infection with *E. coli*, the DIO groups had significantly higher contents of IL-6, leptin, and resistin but lower content of IL-8 compared with the lean groups (*p* < 0.05).

At 12–72 h after infection with *E. coli*, the content of pulmonary TNF-α was significantly higher (*p* < 0.05) in the lean-*E. coli* group compared with the lean-uninfected group. However, in the DIO-*E. coli* group, the content was significantly increased (p < 0.05) only at 24 h compared with the DIO-uninfected group (Fig. 7A).

In the DIO-*E. coli* and lean-*E. coli* groups, the pulmonary IL-1β content was significantly higher (*p* < 0.05) at 12 and 24 h compared with each uninfected group. Moreover, this content was significantly higher in the lean-*E. coli* group than in the DIO-*E. coli* group (*p* < 0.05) at 12 h (Fig. 7B).

The content of IL-6 continuously increased and was significantly higher in the lean-*E. coli* group than in the lean-uninfected group (*p* < 0.05). By contrast, the pulmonary IL-6 content was significantly increased (*p* < 0.05) only at 12 h in the DIO-*E. coli* group compared with the DIO-uninfected group (Fig. 7C).

At 12–72 h after *E. coli* infection, IL-8 content significantly increased (*p* < 0.05) in the *E. coli*-infected groups (Fig. 7D). However, in the lean-*E. coli* group, the fold change was dramatically increased at 12 h and then progressively declined; however, in the DIO-*E. coli* group, it increased continuously.

Leptin and resistin are two typical adipocytokines. At 12–72 h after infection with *E. coli*, the leptin content was significantly increased (*p* < 0.05) in the *E. coli*-infected groups compared with the uninfected groups (Fig. 7E). Moreover, a sharp fold change at 12 h and 24 h was exhibited in the DIO groups and at 72 h in the lean group. The resistin contents showed a completely different tendency between the lean groups and the DIO groups after infection. The resistin contents were significantly increased (*p* < 0.05) at 12 to 72 h in the *E. coli*-infected groups compared with the uninfected groups. The resistin contents in the DIO-*E. coli* group dramatically decreased (*p* < 0.05) compared with the DIO-uninfected group at all time points (Fig. 7F).

After infection with *E. coli*, the regularity of the variation trend in the lean and DIO mice was mainly similar: peaking at 12 or 24 h and then showing a decline. The most changeable factor in the lean mice was TNF-α, whereas that in the DIO mice was resistin. Moreover, the resistin content increased in the lean mice but decreased in the DIO mice.

### Changes on the state of oxidative stress following *E. coli* infection

**Figure 8:**
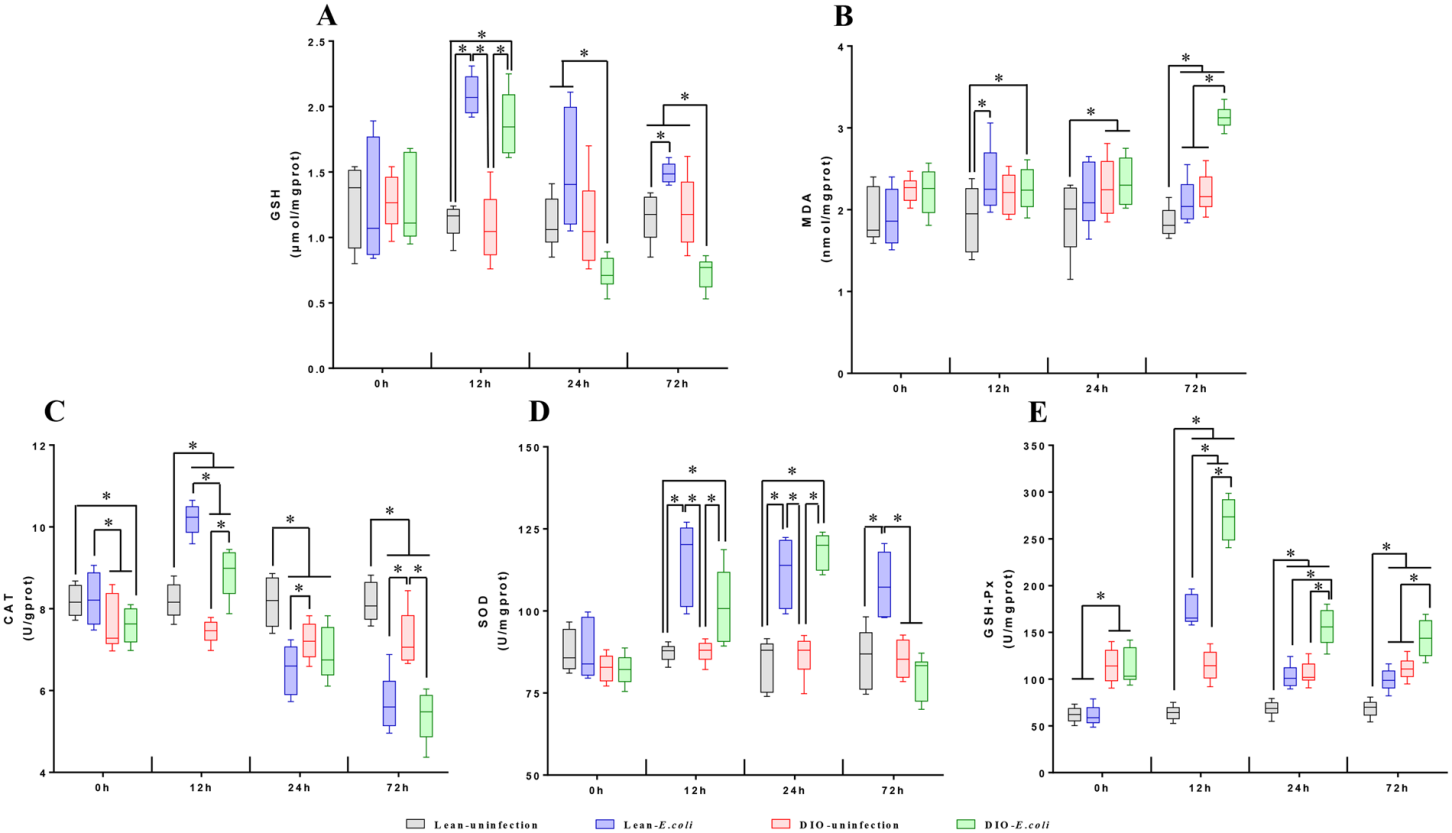
Oxidative stress markers in lung tissue of lean and obese mice following *E. coli* infection. (**A**) Glutathione (GSH), (**B**) Malondialdehyde (MDA) (C-E) Pulmonary redox potential in terms of catalase (CAT), superoxide dismutase (SOD) and glutathione peroxidase (GSH-px) enzyme activities. ^*^p < 0.05.

To evaluate the state of oxidative stress, the contents of glutathione (GSH) and malonaldehyde (MDA) and the activities of GSH peroxidase (GSH-Px), superoxide dismutase (SOD), and catalase (CAT) were measured (Fig. 8). Before infection with *E. coli*, only GSH-Px activity of the DIO groups was evidently higher than that of the lean groups (*p* < 0.05).

After infection, the GSH content significantly increased (*p* < 0.05) at 12 and 72 h in the lean-*E. coli* group compared with the lean-uninfected group. However, in the DIO-*E. coli* group, the GSH content increased only at 12 h (*p* < 0.05) and then decreased from 24 h to 72 h compared with the DIO-uninfected group (Fig. 8A). The MDA content slightly increased in the lean-*E. coli* group compared with the lean-uninfected group at 12 and 72 h, but it dramatically increased in the DIO-*E. coli* group at 72 h compared with the DIO-uninfected group (*p* < 0.05) (Fig. 8B).

CAT activity was markedly increased (*p* < 0.05) in the *E. coli*-infected groups at 12 h after infection and then significantly declined (*p* < 0.05) from 24 h to 72 h in the lean-*E. coli* group and at 72 h in the DIO-*E. coli* group (Fig. 8C). The SOD activity showed an increasing tendency in the lean-*E. coli* group compared with the lean-uninfected group during the whole experiment (*p* < 0.05). The SOD activity significantly increased (*p* < 0.05) in the DIO-*E. coli* group at 12 and 24 h and then showed a decreasing tendency. However, no significance was found (*p* > 0.05) (Fig. 8D). The activities of GSH-Px significantly increased (*p* < 0.05) in the *E. coli*-infected groups from 12 h to 72 h compared with the uninfected groups (Fig. 8E).

In conclusion, the most changeable factors in the lean and DIO mice were GSH and GSH-Px. The lean mice showed more dramatic change than the DIO mice after being infected with *E. coli*. Moreover, in the lean mice, after these parameters markedly increased at 12 h, they declined at 24 h and started to level off from 24 h to 72 h. However, the fluctuation of these parameters in the DIO mice was more irregular.

## Discussion

Obesity is essentially caused by an imbalance in energy intake and expenditure^14^. In accordance with previous studies^15,16^, after feeding with a high-fat diet for 8 weeks, the serum TG and TC levels in the DIO mice were significantly increased compared with those in the lean mice, indicating a higher metabolic reserve in DIO mice. Moreover, in our previous study^17^, a different dosage of *E. coli* was intranasally instilled to establish bacterial pneumonia. Unpredictably, we found that during mild infections (non-fatal dose), obesity improved host defenses against infection, promoting recovery. However, in severe infection (lethal dose), obesity exerted negative effects on host defenses. To understand the protective effect of obesity on lung injury and inflammatory response of mice affecting milder infections, 10^9^ CFUs/mL of *E. coli* was used in the present study.

Metabolic alteration occurs during infection/inflammation, such as increased serum TG and TC levels in rodents^18^. *E. coli* possesses abundant lipopolysaccharide (LPS). The increase in serum TG and TC levels induced by LPS administration may be related to the stimulation of hepatic lipogenesis and adipose tissue lipolysis, lipoprotein lipase activity, decreased TG clearance^19^, and increased low-density lipoprotein cholesterol^20^. Our results also showed that after infection with *E. coli*, the levels of serum TG and TC increased at 12 h in the lean-*E. coli* group, whereas its increase was delayed to 72 h in the DIO-*E. coli* group. These phenomena were similar to pneumonia patients with normal weight who may not have enough metabolic reserve to counteract the increased catabolic stress^21^. Cytokines, especially TNF-α and IL-1β, can acutely increase serum cholesterol and TG metabolism^22^. Interestingly, in the present study, the levels of pulmonary TNF-α and IL-1β, particularly the former, increased sharply in the lean-*E. coli* group compared with the DIO-*E. coli* group at 12 h after infection. Therefore, the dramatic changes in the TG and TC contents in the lean-*E. coli* group may have been, at least partially, attributed to the higher levels of TNF-α and IL-1β than those in the DIO-*E. coli* group.

*E. coli* is one of the main causes for bacterial pneumonia episodes worldwide^23^. Once *E. coli* invades the alveoli, it triggers the immune system to respond by sending white blood cells responsible for attacking microorganisms to the lungs and then releasing cytokines to further activate the immune system^24^. In the present study, after infection with *E. coli*, both the DIO- and lean-*E. coli* groups infiltrated large numbers of inflammatory cells, which led to a significant increase in lung weight and lung index in the *E. coli*-infected groups. Neutrophils are characteristic of acute bacterial suppurative pneumonia, expressing pro-inflammatory cytokines^25,26^. IL-8 is characterized as a neutrophil chemotactic factor in a variety of lung diseases and can cause the recruitment of neutrophils to the pulmonary interstitium and airspace^27^. Alveolar macrophage can destroy bacteria and release various inflammatory cytokines, such as IL-1, IL-6, and TNF-α^28–30^. As revealed by pulmonary cytokine detection, the increase in pulmonary TNF-α, IL-1β, IL-6, and IL-8 levels was more severe in the lean mice than in the DIO mice after infection. This finding was consistent with the severity of inflammatory cell infiltration in histopathological observation. Moreover, as reported previously, mice fed a high-fat diet showed increased vascular permeability and vascular dysfunction^31^, and the chronic low-grade inflammation of obesity impaired pulmonary vascular homeostasis and enhanced susceptibility to acute injury^32^. Our results demonstrated that the DIO mice exhibited higher incidence of congestion before infection and showed more serious hemorrhage after *E. coli* infection than the lean mice, indicating an increased vascular permeability in DIO mice. Therefore, even though the DIO mice were less sensitive to recruit inflammatory cells, they showed more serious damage in pulmonary vasculature than the lean mice.

Elastin and collagen are the major components of the lung connective tissue network, providing the lung with elasticity and tensile strength^1^. The long-term chronic inflammation status in obesity indicates excessive fibrosis deposited in the connective tissue^33^. Pulmonary fibrosis is also associated with increased expression of collagens in connective tissue^34^. Similar to a previous report^33^, our results revealed that DIO mice with high collagen fiber density may increase the risk of pulmonary fibrosis. Moreover, the adventitial connective tissue of bronchioles is primarily loose, with many collagen and elastic fibers oriented longitudinally or perpendicularly to the long axis of the airway^35^, and the wavy structure of collagen fiber provides the lung with a substantial degree of extensibility^1^. In the present study, after infection, inflammatory cells and mucous exudate infiltrated into the adventitial connective tissue of bronchioles and destroyed the wavy structure of collagen fibers in the lung. In addition, by modulating T-cell response, leptin may play a protective role in accumulating pulmonary collagen during inflammation^36,37^. In the present study, the mice in the *E. coli*-infected groups that possessed high levels of leptin exhibited decreased density of pulmonary collagen fibers. Mucins are a family of heavily glycosylated proteins (glycoconjugates) produced by epithelial tissues in most animals, and their key characteristic is to form chemical barriers and bind to pathogens as a part of the immune system^2^. Previous studies detected overexpressed mucins in various lung diseases, such as asthma, bronchitis, chronic obstructive pulmonary disease, and cystic fibrosis^38,39^. Similar to these studies, the secretion of mucins in epithelial cells of the intrapulmonary conducting airways was also increased in the infected groups. Interestingly, the increased secretion of mucins in the lung was greater in the lean-*E. coli* group at 12 and 24 h; however, in the DIO-*E. coli* group it was observed at 72 h.

Obesity is associated with a chronic inflammatory response attributed to the secretion of adipocytokines and various other cytokines^40,41^ and shows complex effects on immune function and inflammation with pro- and anti-inflammatory elements^42^. As previously reported, leptin-deficient mice (*ob*/*ob* mice) exhibit reduced lung neutrophil counts and levels of pro-inflammatory cytokines during pneumonia^43,44^, and obese patients with pneumonia show low pneumonia severity index scores and plasma levels of C-reactive protein^13,45^. Similar to these reports, in our study, the fold change of cytokine contents, especially TNF-α and IL-6, in the lean-*E. coli* group was higher than that in the DIO-*E. coli* group, which might be attributed to increased pulmonary neutrophil and macrophage infiltration in the lean-*E. coli* group after infection. Moreover, obese individuals with high serum cholesterol concentrations can neutralize circulating LPS, therefore decreasing inflammation^46^. Our results revealed high serum TG and TC levels in the DIO mice, which might exhibit lower fold change of adipocytokine and cytokine contents after infection. In addition, resistin is a pro-inflammatory protein and serves as a marker of disease severity and a mediator of prolonged inflammatory state^47^. In the present study, the pulmonary resistin level was down-regulated in the DIO mice, which was one marker of mild infection status, as well as a delayed inflammatory response.

Oxidative stress plays critical roles in the pathogenesis of various diseases. MDA is a marker for oxidative stress and the end product of lipid oxidation^48,49^. GSH-Px is a peroxidase protecting the organism from oxidative damage^50^. Obesity is associated with an increase in oxidative stress and formation of reactive oxygen species (ROS)^51^. Adipocytokines can induce the production of ROS, generating a process known as oxidative stress^51,52^. Based on our results, the DIO mice showed higher MDA contents, GSH-Px activity, and adipocytokine (leptin, resistin, and IL-6) levels than the lean mice, indicating that DIO mice suffered from high oxidative stress levels before infection.

The phagocytosis of gram-negative bacteria can activate the primary host defense mechanism and result in the generation and release of ROS^53^. In our present study, the content of oxidative product (MDA) and antioxidants (GSH) and activity of antioxidase (SOD, CAT, and GSH-Px) were increased in the infected groups at 12 h after infection. Moreover, GSH is an important antioxidant that is capable of preventing damage from ROS under the catalysis of GSH-Px^54^. In this study, the two most increased parameters in the infected group were GSH content and GSH-Px activity, which attempted to prevent the lung from oxidative damage. In addition, obesity can induce the down-regulation of antioxidant enzyme activities^52,55^. To combat infection, different types of cells (e.g., neutrophils and monocytes) rapidly release large numbers of ROS and lysosomes to destroy bacteria^56^. In the present research, the fold change of antioxidant parameter contents was lower in the DIO-*E. coli* group than in the lean-*E. coli* group, which was attributed to the status of obesity and low numbers of infiltrated neutrophils in DIO mice. These antioxidant parameters started to level off from 24 h to 72 h in the lean-*E. coli* group but were still irregular in the DIO-*E. coli* group. Therefore, when the excess lipid oxidation induced by infection continued, obesity-induced oxidoreductase diminution would ultimately cause the increased generation of the lipid peroxide, such as MDA, at 72 h in the DIO-*E. coli* group.

In conclusion, *E. coli*-infection induced an acute suppurative inflammation in the lungs and increased lung weight and lung index, cytokine and adipocytokine levels, and oxidative stress levels in the *E. coli*-infected groups. Interestingly, the DIO mice with high levels of metabolic reserve and adipocytokines exhibited a delayed inflammatory response. Metabolic disorder, neutrophil infiltration, inflammatory factor generation, and increased MDA levels in the lean-*E. coli* group were more important than those in the DIO-*E. coli* group at 12 h; however, these effects were reversed at 72 h. These results indicated that the DIO mice presented a delayed inflammatory response and oxidative stress to the non-fatal acute pneumonia induced by *E. coli* infection.

## Materials and Methods

### Mouse model of diet-induced obesity

A total of 256 male ICR mice (21 days old) were purchased from the Dossy Animal Center (Chengdu, China) and housed under specific-pathogen-free condition. All animal experimental procedures were approved by the Institute of Animal Care and Use Committee at Sichuan Agricultural University (Chengdu, China). The mice received either a normal diet or a high-fat diet, which were both purchased from the Dossy Animal Center. Food and water were supplied *ad libitum*. Based on a previous study^17^, after feeding with high-fat diets for 8 weeks, mice were weighed, and their height was measured. Mice with obese index exceeding 20% and significantly high Lee’s index were considered as obese animals.

$$\begin{array}{rcl}{\rm{Obese}}\,{\rm{index}} & = & \frac{{\rm{Individual}}\,{\rm{weight}}\,{\rm{of}}\,{\rm{DOI}}-{\rm{Average}}\,{\rm{weight}}\,{\rm{of}}\,{\rm{Lean}}}{{\rm{Average}}\,{\rm{weight}}\,{\rm{of}}\,{\rm{Lean}}}\times 100 \% \\ \mathrm{Lee}\mbox{'}s\,{\rm{index}} & = & \frac{\sqrt[3]{{\rm{Body}}\,\mathrm{weight}\,\,({\rm{g}})\times {10}^{3}}}{{\rm{Body}}\,\mathrm{length}\,\,({\rm{cm}})}\times 100 \% \end{array}$$

All experimental procedures were performed in accordance with the national and international guidelines and regulations and were approved by Sichuan Agricultural University Animal Care and Use Committee (Approval No: 2012-024).

### Mouse model of acute pulmonary infection

*E. coli* obtained from Veterinary Medical Laboratory of Sichuan Agricultural University (Ya’an, China) was cultured in Luria-Bertani broth (5 g of yeast extract, 10 g of tryptone, 10 g of NaCl/L) at 37 °C for 18 h. Then, the bacterial culture was centrifuged, and bacterial pellets were resuspended in phosphate-buffered saline (PBS) to produce the inoculums.

After 8 weeks, the mice fed with either normal or high-fat diets were divided into two groups (128/group) as lean and DIO groups, respectively. Then, the two groups were subdivided into four groups (64/group) after being intranasally instilled with PBS or *E. coli*, namely, lean-uninfected, DIO-uninfected, lean-*E. coli*, and DIO-*E. coli* groups.

The mice in the lean-*E. coli* and DIO-*E. coli* groups were quickly anesthetized with ether and intranasally instilled with 40 μL of *E. coli* inoculum (containing approximately non-fatal 4 × 10^9^ colony-forming units) suspended in PBS according to a previous study^17^. The same amount of PBS was given to the mice in the lean-uninfected group and DIO-uninfected group by the same method. Animals were monitored closely for 72 h after *E. coli* infection. After intranasal infection with *E. coli* or PBS, mice were sacrificed under anesthesia at 0 (pre-infection), 12, 24, and 72 h (post-infection), and the lungs were harvested and prepared for the following analyses.

### Lung index measurement

During the experiment, no mouse died after *E. coli* infection. After necropsy, the lungs were collected without connective tissues and weighed. The lung index was calculated using the following formula:

$${\rm{Lung}}\,{\rm{index}}=\frac{{\rm{Lung}}\,\mathrm{weight}\,\,({\rm{g}})}{{\rm{Body}}\,\mathrm{weight}\,\,({\rm{g}})}\times 100 \% $$

### Serum triglycerides and total cholesterol determination

Blood samples were obtained retro-orbitally from eight mice in each group, and individual sera were separated via centrifugation and stored at −20 °C. TG and TC concentrations in the serum were determined using a commercially available kit (A110-1 and A111-1, Nanjing Jiancheng Bioengineering Institute, China).

### Histopathological assessment of lung injury

Eight mice from each group were euthanized. The lungs were observed and photographed and then immediately fixed in 4% paraformaldehyde. Subsequently, they were dehydrated in alcohol, embedded with paraffin, sectioned at 5 μm, and processed for hematoxylin and eosin staining and the following specific histological stainings: Masson’s trichrome for fiber and AB-PAS for mucins. Histopathological changes were observed and photographed with a digital camera (Nikon DS-Ri1, Japan). By Masson’s trichrome staining, collagen fiber stains blue, erythrocyte stains red, and cell nucleus stains black. By AB-PAS staining, neutral mucins stain red and acid mucins stain blue. The same position of the lungs in eight mice was observed through a microscope, and the histological lesion of the lung was evaluated through the incidence of congestion and hemorrhage, severity scoring of neutrophil infiltrate, and proportion of airspace areas. The level of severity was judged from − to ++++, which represented none to severe. The airspace proportion was the ratio of the airspace area and the total area of one view under 400× magnification.

The total number of bronchioles in eight mice was counted through a microscope, and the number of bronchioles that contained neutral or acid mucins was recorded. The occurrence rate of mucins (neutral or acid) was the ratio of the mucin-stained bronchiole number and the total bronchiole number, which was calculated to reflect the mucin content in the lung. The Integrated Optical Density (IOD) of collagen fibers was measured to represent the collagen fiber contents of lung. The airspace area proportion and IOD of collagen fibers were analyzed using image-Pro Plus 6.0 (USA).

### Lung tissue cytokine measurement by Enzyme-Linked Immunosorbent Assay (ELISA)

At the indicated time points, eight mice in each group were euthanized, and the lungs were immediately collected for evaluating the contents of several cytokines and adipocytokines. The lung (1 g) was homogenized with normal saline buffer (9 mL) through a cell homogenizer in an ice bath and centrifuged at 3,000 r/min for 10 min to obtain a clear supernatant. TNF-α, IL-1β, IL-6, IL-8, leptin, and resistin concentrations in pulmonary supernatants of mice in each group were measured with mouse ELISA kits (H052, H002, H007, H008, H174 and H175, Nanjing Jiancheng Bioengineering Institute, China) according to the manufacturer’s instructions.

### Lipid peroxidation and antioxidant defense system assays

After determining the concentration of total protein in the supernatant of the pulmonary homogenate by the bicinchoninic acid method, the GSH and MDA contents and GSH-Px, (SOD), and CAT activities were measured by biochemical method following the instruction of reagent kits (A006-2, A003-1, A005, A001-3 and A007-1, Nanjing Jiancheng Bioengineering Institute, China). The GSH assay was based on the development of a yellow color when 5,5′-dithio-bis-nitrobenzoic acid was added to compounds containing sulfhydryl groups. The MDA assay was determined by the thiobarbituric acid colorimetric method. The GSH-Px activity was detected by the consumption of GSH. The SOD activity was determined by the xanthine oxidase method. The CAT activity was determined by the H_2_O_2_ decomposition rate. The values were expressed as nmol/mg protein for GSH and MDA and units (U)/mg or U/g of protein for GSH-Px, SOD, and CAT.

### Statistical analysis

The significance of difference between two groups was analyzed by the independent samples *t* test, whereas the significant differences among four groups within 72 h experiment were analyzed by variance analyses (LSD or Dunnett’s T3). The results were expressed as means ± standard deviation. The analyses were performed using SPSS 17.0 software (IBM Corp, Armonk, NY, USA) for Windows. Statistical significance was considered at *p* < 0.05.
